# Impact of Aquaculture Practices on Intestinal Bacterial Profiles of Pacific Whiteleg Shrimp *Litopenaeus vannamei*

**DOI:** 10.3390/microorganisms7040093

**Published:** 2019-03-30

**Authors:** Angela Landsman, Benoit St-Pierre, Misael Rosales-Leija, Michael Brown, William Gibbons

**Affiliations:** 1 trū Shrimp Innovation Center, The trū Shrimp Company, 330 3rd Street, Balaton, MN 56115, USA; Misael.Rosales@trushrimpcompany.com; 2 Department of Biology and Microbiology, South Dakota State University, Alfred Dairy Science Hall, Box 2104A, 1224 Medary Avenue, Brookings, SD 57007, USA; William.Gibbons@SDState.edu; 3 Department of Animal Science, South Dakota State University, Animal Science Complex, Box 2170, Brookings, SD 57007, USA; Benoit.St-Pierre@SDState.edu; 4 Department of Natural Resource Management, South Dakota State University, Edgar S. McFadden Biostress Lab, Box 2140B, 1390 College Avenue, Brookings, SD 57007, USA; Michael.Brown@SDState.edu

**Keywords:** aquaculture, intestinal microbiome, Pacific whiteleg shrimp

## Abstract

Considering the crucial role of the gut microbiome in animal health and nutrition, solutions to shrimp aquaculture challenges, such as improving disease resistance and optimizing growth on lower cost feeds, may lie in manipulation of their microbial symbionts. However, achieving this goal will require a deeper understanding of shrimp microbial communities and how their composition is influenced by diet formulation, environmental conditions, and host factors. In this context, the current study investigated the intestinal bacterial communities of the Pacific whiteleg shrimp (*Litopenaeus vannamei*—the most widely aquaculture-farmed shrimp worldwide) reared in indoor aquaculture facilities and outdoor pond systems. While samples showed very consistent intestinal bacterial community profiles within each production system, major differences were uncovered between the two practices. Indeed, bacteria affiliated with Rhodobacteraceae (Proteobacteria) and Actinobacteria were significantly more abundant in indoor samples (84.4% vs. 5.1%; 3.0% vs. 0.06%, respectively), while Vibrionaceae (Proteobacteria), Firmicutes, Fusobacteria and Cyanobacteria were predominant in pond samples (0.03% vs. 44.8%; 0.7% vs. 36.0%; 0.0% vs. 7.9%; 0.001% vs. 1.6%, respectively). Accordingly, the abundance of 11 of the 12 most prominent Operational Taxonomic Units (OTUs) were found to be statistically different between the two production environments. Together, these results indicate that aquaculture practices greatly influence the intestinal bacterial profile of the whiteleg shrimp, and further suggest that bacterial communities of this economically important crustacean could be effectively manipulated using diet composition or environmental conditions.

## 1. Introduction

Shrimp is one of the most important seafood traded worldwide, with more than 3.4 million tons marketed each year at an estimated wholesale price ranging between $3800 and $8800 USD per ton [1]. As the global human population continues to grow, shrimp supplies will need to double in the next 20 years in order to meet future demand [2]. As wild harvest capture has grown stagnant [3], aquaculture has become the most viable alternative to meet current and future shrimp market demands [2]. Indeed, 55% of the annual global shrimp supply in 2018 was produced by farming [4], suggesting that aquaculture has the capacity to provide consumers with a consistent and reliable supply of product [5]. While still in its infancy, shrimp farming has shown great potential for high productivity at reduced costs. For instance, aquaculture-raised shrimp have shown twice the growth rates of wild stocks, indicating great potential to further increase production [6]. Due to its tolerance to a wide range of salinities and temperatures, whiteleg shrimp (*Litopenaeus vannamei*), also known as Pacific white shrimp or king prawn, is the most widely farmed shrimp worldwide [7,8].

Outdoor ponds with close access to ocean water represent the most popular and basic design for shrimp farming. Regular exchanges with ocean water are used to both replenish food sources for growing shrimp and to evacuate waste from the ponds. Variations amongst production systems are typically a function of stocking density, which primarily affects ocean water inputs needed to maintain water quality [9]. Since they require minimal inputs to ensure growth, ponds represent an attractive, low-cost production system [10]. However, the impact of pond-based production on the environment, as well as risks for pathogen outbreaks, are cause for concern. Effluent from shrimp production ponds is a significant source of chemical and biological pollutants in ocean waters that can harm natural aquatic habitats that are sensitive to excessive nutrient loads [2,5]. The exposed nature of open ponds means they typically have limited protection against exposure to pathogens, with high density ponds at greater risk of experiencing outbreaks as a result of increased pollution and stress conditions [6]. Heavy losses in shrimp production due to disease have historically been a defining feature of this industry [11], particularly from pathogens of the *Enterobacteriaceae* family, which include species affiliated to *Pseudomonas*, *Flavobacterium*, *Escherichia*, *Aeromonas*, *Vibrio*, *Rickettsia*, *Shewanella* and *Desulfovibrio* [12]. Once infected, ponds themselves present a risk for contaminating wild populations residing in nearby native waters [11]. Treatment of pond water with antibiotics is a common strategy to mitigate risk of disease, but this practice can lead to the selection of resistant microbial strains that could be transferred to the human food supply [13], as well as surrounding natural waters. The trend for stricter antibiotic regulations worldwide in livestock production also indicates that alternative methods in aquaculture will be needed in the near future.

In contrast to ponds, indoor facilities for shrimp farming allow tighter biosecurity control and help lower the environmental footprint of aquaculture. While they require more costly investments in infrastructure, such as recirculating water systems, indoor facilities can dramatically minimize exposure to environmental pathogens, provide better control of water quality with reduced impact on the environment, and provide safer seafood products that are free from food-borne contaminants [2]. Besides facility costs, the other main disadvantage of indoor aquaculture is the need to formulate diets that optimize shrimp growth at reduced costs. Ingredients have traditionally been derived from fish byproducts such as fish meal and fish oils, but the higher cost of these feedstuffs and other animal protein sources has motivated the use of plant-derived proteins [12,14]. However, because plant products are not natural components of shrimp diets, and contain high levels of carbohydrates and anti-nutritional factors that shrimp have not evolved to digest, high inclusion levels may result in sub-optimal growth and poor health [12].

Considering the overarching importance of the intestinal microbiome in animal health and nutrition [15], manipulation of beneficial microbial communities in the shrimp gut may provide solutions to improve resistance to pathogens without prophylactic use of antibiotics, as well as to optimize growth on alternative protein sources. Research to date has found that the gut bacterial profile in healthy shrimp consists primarily of Proteobacteria, consistent with marine fish [16], which is in stark contrast to the microbiome of terrestrial animals in which Firmicutes and Bacteroides are typically dominant [17]. Indeed, these latter phyla have so far been found to be minor components of the shrimp gut microbiome, and their abundance appears to be highly dependent on local environmental conditions and diet composition [12]. There is limited knowledge to date on the effect of aquaculture practices on the shrimp gut microbiome [15]. For instance, biosafety measures to prevent pathogen outbreaks may also inadvertently reduce or prevent colonization of indoor raised shrimp with beneficial bacteria found in natural environments [15]. Since early gut microbial colonization events can impact the future performance or productivity of an animal, proper microbiome development may be detrimentally altered in aquaculture raised shrimp [14]. Conversely, gut bacterial communities of shrimp have been shown to adapt to commercially formulated diets, indicating that the shrimp microbiome can be manipulated through dietary ingredients [14].

Since diet and host genetics, as well as a number of environmental conditions such as water temperature, salinity, and sulfide concentrations, can affect the gut microbiome composition of whiteleg shrimp [12], we hypothesized that the intestinal bacterial communities of this highly farmed aquatic species would differ between the two main types of aquaculture systems. To this end, the study presented in this report compared the intestinal bacterial profile of whiteleg shrimps raised in an indoor facility with individuals of the same species collected from two pond systems. Within each production system, which were operated under their respective normal practices, samples showed very consistent taxonomic profiles and gut bacterial community structures. However, major differences in taxonomic affiliations and Operational Taxonomic Unit (OTU) profiles were uncovered between shrimp raised in an indoor facility and shrimp raised in ponds. Together, these results indicate that aquaculture farming conditions greatly influence the composition of the white-leg shrimp gut microbiome.

## 2. Materials and Methods

### 2.1. Sample Collection and Harvesting of Shrimp Intestinal Tissue

Shrimp were captured from three different production environments (as described below). Intestinal tissue was harvested from each animal using the following procedure. The telson was removed with scissors distal to the sixth abdominal segment, then the posterior end of the carapace was lifted to expose the hepatopancreas and the proximal end of the gut. The intestine was then excised with sterile tweezers starting at the hepatopancreas on through to the hind gut. Each sample consisted of intestinal tissue pooled from five individual shrimp from the same population (see description below) to ensure sufficient material was available for DNA extraction. All harvested intestinal tissue samples were stored at −20 °C until DNA extraction.

### 2.2. Study Site for the Indoor-Raised Shrimp

The trū Shrimp Innovation Center (330 3rd Street, Balaton, MN, USA; 44.2° N 95.8° W) is a research campus designed to industrialize indoor aquaculture techniques for shrimp production. The facility contains nearly 200 clearwater and biofloc research tanks, as well as other commercial production tanks of various sizes and configurations. Indoor-raised shrimp were maintained at 28 ± 1 °C in temperature-controlled tanks. Water management was carried out using separate indoor recirculating aquaculture systems, one for each tank, utilizing fresh water processed by reverse osmosis then mixed at 28 g Marinemix (Marine Enterprises International, LLC., Baltimore, MD, USA) to one-liter production water. Total ammonia nitrogen (TAN) levels were maintained at less than 3.0 mg/mL, nitrite levels below 4.5 mg/mL, and nitrate levels never exceeded 100 mg/mL. Evaporated water was replaced with fresh water as needed to maintain salinity at 28 ppt. Stocking densities were maintained in the standard range of intensive production systems at 30–60 shrimp per cubic meter, with feed offered continuously. All culture tanks were supplemented with a commercial blend of probiotic bacteria (BioWish 3P, BioWish Technologies, Cincinnati, OH, USA) containing *Pediococcus acidilactici*, *P. pentosaceus*, *Lactobacillus plantarum* and *Bacillus subtilis*. The probiotic was provided as a daily dose of 0.025 g/100 L, which was manually administered over the course of a 24-h period.

Fourteen healthy indoor-raised aquaculture samples were obtained from six production tanks fed three separate diets at three different time points (see Table 1 for sample description). The diets fed all contained 35% crude protein, and consisted of Production 35% (Rangen Inc., Buhl, ID, USA), Hyper-Intensive 35 (Zeigler Bros. Inc. Gardners, PA, USA) and tSC (tSC 35%, tSC Grow Out, Balaton, MN, USA). Data from partial nutrient composition of diets used is presented in Table 2. As they did not require long distance transportation, no preservative was added to intestinal tissue samples from indoor-raised shrimp, and they were immediately stored at −20 °C after dissection.

### 2.3. Pond-Raised Shrimp

Samples from healthy, pond-raised shrimp were obtained from two farms. Ten samples were collected from a shrimp farm located on the coast of Kino Bay (Sonora, Mexico—samples labeled ‘KB-’), and five other samples were obtained from a farm located near Obregon (Sonora, Mexico—samples labeled ‘Ob-’). Ponds averaged a depth of 1.5 meters, with stocking densities maintained within a range typical of intensive production systems (30–60 shrimp/m^3^). Water chemistry testing was conducted weekly to monitor levels of TAN, nitrite, nitrate and alkalinity. These parameters were used to determine the rates of water exchange to maintain water quality, which ranged from 0% to 20%. Both sites fed the same commercial diet (Bumper Crop, Vimifos S.A. de C.V., Guadalajara, Jalisco, Mexico; data from partial nutrient composition is presented in Table 2), with the Obregon farm ponds receiving a daily dose of phytochemicals as a dietary supplement. Feed was offered at scheduled times during the day (5 a.m., 12 p.m. and 5 p.m.). Pond sampled shrimp were harvested at approximately day 80 of age, then dissected on site. Intestinal samples were preserved in 100% ethanol for transportation, and then stored at −20 °C upon arrival at the host laboratory.

### 2.4. Wild-Caught Shrimp

It is very challenging to obtain wild-caught shrimp that are not degraded from storage or preservation methods on a fishing vessel. Therefore, only one wild-caught sample (five shrimp from the same catch) of high quality could be obtained for this study. These shrimp of undetermined age and natural diets were caught in the Gulf of California (34 to 36 ppt salinity). Dissected intestinal samples were preserved in 100% ethanol for transportation, then stored at −20 °C upon arrival at the host laboratory. Because a single sample increases the risk of potential bias, the microbiome composition of the wild caught sample was only used as a qualitative reference.

### 2.5. Microbial DNA Isolation and PCR Amplification

Microbial DNA was isolated from shrimp intestinal samples using the repeated bead beating plus column method, as described by Yu and Morrison [18]. The V1–V3 region of the bacterial 16S rRNA gene was PCR-amplified using the 27F forward [19] and 519R reverse [20] primer pair. PCR reactions were performed with the Phusion Taq DNA polymerase (Thermo Scientific, Waltham, MA, USA) under the following conditions: hot start (4 min, 98 °C), followed by 35 cycles of denaturation (10 s, 98 °C), annealing (30 s, 50 °C) and extension (30 s, 72 °C), then ending with a final extension period (10 min, 72 °C). PCR products were separated by agarose gel electrophoresis, and amplicons of the expected size (~500 bp) were excised for gel purification using the QiaexII Gel extraction kit (Qiagen, Hilden, Germany). For each sample, approximately 400 ng of amplified DNA were submitted to Molecular Research DNA (MRDNA, Shallowater, TX, USA) for sequencing with the Illumina MiSeq 2X300 platform to generate overlapping paired end reads.

### 2.6. Computational Analysis of PCR Generated 16S rRNA Amplicon Sequences

Unless specified, sequence data analysis was performed using custom written Perl scripts (available upon request). Raw bacterial 16S rRNA gene V1–V3 amplicon sequences were provided by Molecular Research DNA as assembled contigs from overlapping MiSeq(2X300) paired-end reads from the same flow cell clusters. Reads were then selected to meet the following criteria: presence of both intact 27F (forward) and 519R (reverse) primer nucleotide sequences, length between 400 and 580 nt, and a minimal quality threshold of no more than 1% of nucleotides with a Phred quality score lower than 15.

Following quality screens, sequence reads were aligned, then clustered into Operational Taxonomic Units (OTUs) at a genetic distance cutoff of 5% sequence dissimilarity [21]. While 3% is the most commonly used clustering cutoff for 16S rRNA, it was originally recommended for full length sequences, and may not be suitable for the analysis of specific subregions since nucleotide sequence variability is not constant across the entire length of the 16S rRNA gene. In this context, if 3% is a commonly accepted clustering cutoff for V4 or V4–V5 regions, which are the least variable of the hypervariable regions, then a higher cutoff should be used for the V1–V3 region, since V1 is the most variable region of the 16S rRNA gene. These and other considerations on the use of 5% as an OTU clustering cutoff can be found in Supplementary Materials and Methods. OTUs were screened for DNA sequence artifacts using the following methods. Chimeric sequences were first identified with the chimera.uchime and chimera.slayer commands from the MOTHUR open source software package [22]. Secondly, the integrity of the 5′ and 3′ ends of OTUs was evaluated using a database alignment search-based approach; when compared to their closest match of equal or longer sequence length from the NCBI nt database, as determined by BLAST [23], OTUs with more than five nucleotides missing from the 5′ or 3′ end of their respective alignments were discarded as artifacts. Finally, single read OTUs were removed from the analysis.

After the removal of sequence chimeras, artifacts, and singleton OTUs, taxonomic assignment of OTUs was determined using a combination of RDP Classifier [24] and BLAST [23]. The List of Prokaryotic Names with Standing in Nomenclature (LPSN http://www.bacterio.net) was also consulted for information on valid species belonging to taxa of interest [25].

### 2.7. Computational Analysis for Alpha and Beta Diversity

Using custom Perl scripts, each dataset was randomly rarefied to 5000 reads, with the 50 iterations created for each sample used to create ‘shared’-type formatted files. All subsequent steps were performed using commands in MOTHUR [22]. Chao1, Shannon and Simpson indices, as well as observed OTUs and coverage, were determined from the shared files using summary.single. Rarefaction curves can be found in Supplementary Figure S1. For Principal Coordinate Analysis (PCoA), Bray-Curtis distances were first determined using summary.shared, which were then used as input for the command pcoa. Principal Components 1 (PC1) and 2 (PC2), representing the highest level of variation, were plotted using Microsoft® Excel. Hierarchical cluster analysis was performed using the online tool iDEP.81 (http://bioinformatics.sdstate.edu/idep) to produce a visual heatmap.

### 2.8. Statistical Analysis

An independent t-test was used to compare the relative abundance of bacterial taxonomic groups and OTUs between different production systems, respectively (GraphPad Software, https://www.graphpad.com/quickcalcs/ttest1.cfm). The means of two groups were considered to be significantly different when *P* ≤ 0.05.

### 2.9. Accession Numbers for Next Generation Sequencing Data

Raw sequence data are available from the NCBI Sequence Read Archive under Bioproject PRJNA522274 and SRA accession SRP185856. Accession numbers for individual samples are provided in Supplementary Table S1.

## 3. Results

### 3.1. Comparative Analysis by Taxonomic Composition

A total of 699,259 high quality and chimera/artifact-free reads were used for analysis, with an average of 22,698 ± 1744 reads per sample for the indoor-raised shrimp samples, 24,796 ± 4223 reads per sample for the pond samples, and 9539 reads for the wild-caught sample. A total of 6988 sequence reads, ranging between 22 and 1025 reads per sample, were designated as ‘unclassified’ because they could not be assigned to a known phylum. 

**Figure 1:**
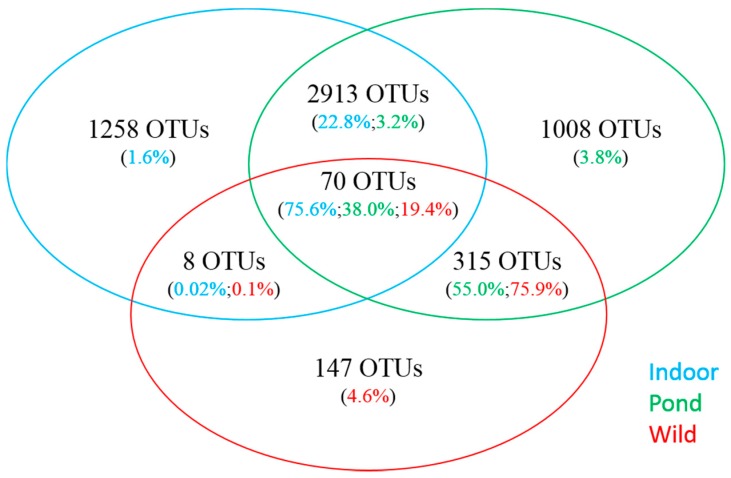
Venn diagram showing the number of shared and unique OTUs from the intestine of indoor-raised, pond-reared and wild caught white leg shrimp. Also shown is the proportion of sequence reads for each category.

Gut bacterial communities of indoor- and pond-raised whiteleg shrimp were found to be very different (*P* < 0.05) in taxonomic profiles. Proteobacteria was overall the most dominant phylum across the samples analyzed, but significantly higher levels were found in indoor samples (88.6%) compared to ponds (51.8%) (Table 3). More discernable differences were observed between the two aquaculture environments at the family level, as bacterial communities from indoor samples were primarily composed of members of the Rhodobacteraceae family (84.4%), while Vibrionaceae were found to be the most abundant in pond samples (44.8%). In addition, Firmicutes, Fusobacteria and Cyanobacteria were found in much higher abundance in pond samples (36.0%, 7.9% and 1.6%, respectively) compared to indoor samples (0.7%, 0.0% and 0.001%, respectively), in contrast to Actinobacteria, which were more highly represented in indoor samples (3.0% vs. 0.06%). While the limited number of wild-caught samples did not allow a statistically-based comparison, a qualitative assessment of abundance values revealed that the overall taxonomic composition of wild-caught shrimp was more similar to pond-raised shrimp than shrimp farmed in indoor facilities (Table 1, Figure 1).

### 3.2. Comparative Alpha and Beta Diversity Analyses

**Figure 2:**
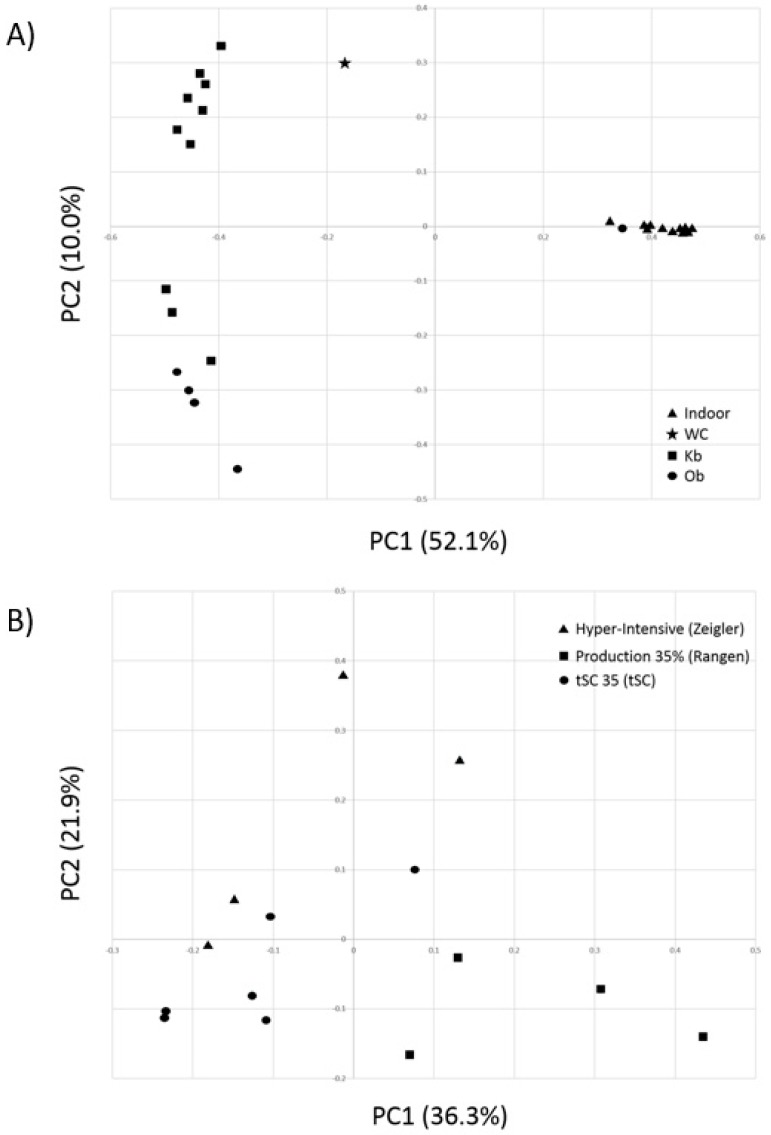
Comparison of intestinal bacterial communities from whiteleg shrimp using Principle Coordinate Analysis (PCoA). (**A**) Comparative analysis between shrimps raised under two different production systems and from one wild population. (**B**) Comparison amongst samples from white-leg shrimp raised in an indoor system under three different diets. The x and y axes correspond to Principal Components 1 (PC1) and 2 (PC2), which explained the highest level of variation.

**Figure 3:**
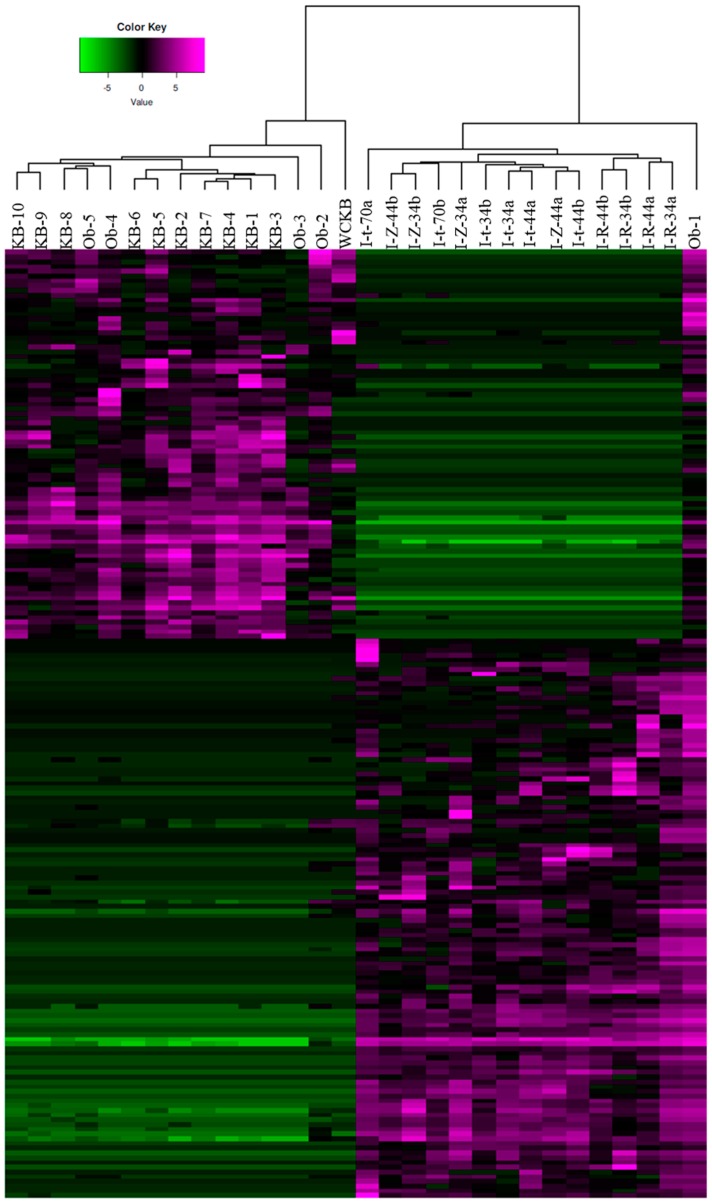
Hierarchical cluster analysis based on the 200 most abundant OTUs from the intestinal bacterial communities of white-leg shrimp.

To further explore differences in bacterial community composition between the shrimp farming environments investigated, we conducted an alpha diversity analysis. Gut bacterial communities of indoor and pond-farmed shrimp were not found to be statistically different in terms of number of observed OTUs or diversity indices such as chao1, ace, Shannon or Simpson (Table 4). However, PCoA using Bray-Curtis distances based on OTU compositional dissimilarity showed clear differences between the intestinal bacterial communities of shrimp raised indoor and those from pond-farmed shrimp (Figure 2). With the exception of the Ob-1 sample, there was a clear separation between the respective sets of samples. Similarly, hierarchical cluster analysis also indicated that indoor and pond samples (with the exception of the Ob-1 sample) grouped separately based on their OTU composition (Figure 3).

### 3.3. Comparative Analysis of Prominent OTUs

To gain further insight, the most abundant bacterial OTUs identified in this study were investigated in more detail (a complete OTU table is provided in Supplementary Table S2). As expected from the taxonomic composition and beta diversity analyses presented above, the profile of main bacterial OTUs from the gut of indoor-raised shrimp was very different from pond-farmed shrimp, with the abundance of 11 of the 12 most prominent OTUs found to be statistically different between the two environments (Table 5). The four prominent OTUs of indoor-raised shrimp (SD_Shr-00001, SD_Shr-00002, SD_Shr-00006, and SD_Shr-00009) were affiliated to the family Rhodobacteraceae (phylum Proteobacteria), together representing on average 72.2% of identified bacteria in these samples, in contrast to 4.1% in pond-raised shrimp. Six other main OTUs were dominant in pond-raised shrimp, and they were affiliated to Vibrionaceae of the phylum Proteobacteria (SD_Shr-00004 and SD_Shr-00005), Firmicutes (SD_Shr-00003 and SD_Shr-00008), as well as Fusobacteria (SD_Shr-00007 and SD_Shr-00015). Interestingly, five of the main pond OTUs were not detected in indoor-raised shrimp. Wild-caught shrimp showed a distinct OTU profile as well, with predominance of SD_Shr-00005 and SD_Shr-00046, which were found in much higher abundance in this sample compared to either indoor or pond-raised shrimp. All Proteobacteria-affiliated main OTUs were found to be very closely related to a known species, with sequence identity values ranging between 98.5% and 99.6%, indicating that each may have represented a strain of its respective closest relative. Conversely, two of the Firmicutes-affiliated OTUs that were prominent in pond-raised shrimp (SD_Shr-00003 and SD_Shr-00008) showed very limited identity to their respective closest validated taxon, indicating that they may have corresponded to members of a bacterial phylogenetic lineage that is yet to be characterized.

## 4. Discussion

In this study, the intestinal bacterial communities of whiteleg shrimp raised under different aquaculture production systems were investigated. Considering the paucity of data available from indoor-raised shrimp, samples were collected from animals fed different diets at various stages of their development in an effort to be representative of potential variability in bacterial profiles. Remarkably, indoor-raised samples showed very homogenous gut bacterial taxonomic composition and community structures regardless of diet and growth stage, as best shown by PCoA analysis (Figure 2). While gut bacterial profiles from pond-raised shrimp were not as closely related to each other as indoor samples, they were consistent as a group and very distinct from those of indoor-raised shrimp. Notably, 11 of the most abundant OTUs were significantly different between the two types of samples, suggesting an effect of production systems on the intestinal bacterial communities of the whiteleg shrimp.

In terms of phylogeny, genetics and metabolic potential, bacteria typically represent the most diverse group of microorganisms in animal gut environments. The relationship between a host and its symbiotic bacteria is the result of co-evolution between the two entities, as mutually beneficial adaptations are selected to favor their association [12]. The development of gut microbial communities in hatchling or neonatal animals involves successive waves of colonization and succession, concurrent with the development, maturation, and food habits of their host. The development of the intestinal microbiome in whiteleg shrimp starts during the fifth nauplius stage, as movement of fluid through the gut is initiated by the anal pore, later followed by major changes at mysis and early post larval stages that occur as a result of feeding on invertebrates such as Artemia and *Rotifera* spp. after hatching [12]. Gut bacterial communities during all developmental stages consist primarily of Proteobacteria, Bacteroidetes and Actinobacteria [14], with representation of each group fluctuating in response to diet changes and development of their host. Other factors, such as salinity, stress, host immune response, exposure to antibiotics, and environmental conditions can affect the composition of gut microbial communities [15]. Consequentially, these factors have a great influence on the ability of gut microbiomes to contribute to the health and nutrition of their host prior and during the productive stages of their life cycle. While many of the mechanisms involved remain to be resolved, a wide body of research focused on humans and animal models has indicated that the type of microorganisms that a young animal is exposed to can affect the composition of its microbiome as it matures.

In natural aquatic systems, shrimp are exposed to microorganisms from water and sediments which provide a pool or source of potential symbionts that can colonize their intestinal tract at various stages of their development [15]. Indeed, at least ninety genera have been found to be shared amongst pond sediments, pond water and the shrimp intestinal tract, with the most similarities found between sediment and gut profiles [26]. Because *Lactobacillus*, *Streptococcus* and *Bacillus* are the most abundant shared genera in the shrimp intestinal tract (1.0%, 0.93% and 0.37%, respectively) [12], and that they are already used as probiotics in humans and other food animals, they have been a major source of probiotics in the shrimp aquaculture industry. While their abundance tends to be low (<1%) in the shrimp intestinal environment, their use as probiotics has been found to be beneficial to the immunity of the host and its efficiency in digesting commercial diets [14]. In this current study, however, they were found at much lower levels, with *Lactobacillus* identified in only one pond shrimp sample (0.01%), *Streptococcus* in three indoor shrimp samples and one pond shrimp sample (0.02% or less), and *Bacillus* not detected in any of the shrimp samples analyzed. However, it is possible that these probiotic species could affect the composition of bacterial communities in the gut of indoor-raised shrimp even if they do not appear to become established in that environment. As suggested elsewhere [12], perhaps probiotic formulations for shrimp production should include beneficial bacterial strains that are native to the shrimp gut.

A number of animal health problems in shrimp can be caused by poor nutrition, as it leads to undeveloped or weak individuals that become more susceptible to disease [17]. The risk for such nutrition problems may be higher with commercial dietary formulations, since the industry is transitioning from fish meal and oils to plant-based ingredients in order to reduce operating costs [12]. Even though the shrimp sampled from indoor production tanks were sourced from distinct tank populations, had independent water sources and management, and were each fed one of three dietary treatments, their gut bacterial profiles were remarkably similar (Figure 1 and Figure 2). Because the indoor diets were formulated using varying combinations of animal and plant-derived protein sources, these results would indicate that while shrimp gut bacterial profiles are highly influenced by dietary ingredients, as shown by the comparison between indoor-raised and pond-raised shrimp in this report, bacterial composition may not be as sensitive to variations in formulations with the same set of ingredients. Plant-derived protein sources in commercial shrimp diets tend to also include polysaccharides and anti-nutritional factors, which the shrimp intestinal tract and natural microbiome have not evolved to digest effectively. Interestingly, while members of the phylum Bacteroidetes are typically considered to be the main utilizers of plant polysaccharides in most gut environments, their abundance was not found to vary significantly amongst the different shrimp production environments investigated (Table 3). While future investigations will be required to elucidate the mechanisms involved, perhaps bacteria from other phyla allow farmed shrimp to effectively use plant-derived protein ingredients. Likely candidates would include SD_Shr-00001, SD_Shr-00002, SD_Shr-00006, and SD_Shr-00009, which were OTUs affiliated to Rhodobacteraceae that were much more abundant in indoor-raised shrimp compared to pond or wild-caught populations. Intriguingly, Firmicutes have been reported in higher abundance in the gut of whiteleg shrimp fed corn starch compared to shrimp fed glucose and sucrose [27].

In addition to diet, exposure to stress or other perturbations can disrupt composition of gut microbiomes resulting in a state of dysbiosis which can provide an opportunity for pathogenic bacteria to thrive, leading to disease [17]. During the shrimp production cycle, stress can be induced from overcrowding or changes in water salinity and quality [12]. While their effect may be undetectable under normal farming conditions, opportunistic pathogens are thought to be ubiquitous in the microbiome of healthy individuals [14]. The main bacterial pathogens that can infect shrimp include members of the genera *Pseudomonas*, *Flavobacterium*, *Escherichia*, *Aeromonas*, *Vibrio*, *Rickettsia*, *Shewanella* and *Desulfovibrio* [12]. Amongst these, *Vibrio*-affiliated sequences were by far the most abundant in this study, particularly OTU SD_Shr-00004, with a mean abundance of 26.8% in pond-samples (Table 4). In contrast, no sequences affiliated to *Flavobacterium*, *Escherichia*, *Aeromonas* or *Rickettsia* were identified amongst the 30 samples analyzed, while sequences for *Desulfovibrio* (0.02% or less in three pond samples, 0.3% in the wild-caught sample), *Shewanella* (0.003% or less in two pond samples) and *Pseudomonas* (one indoor and one pond sample at 0.003% or less, respectively) were only found at very low representation. Notably, SD_Shr-00004 was very closely related to *Vibrio alginolyticus*, an opportunistic pathogen that can cause disease under stress conditions [28,29]. *V. alginolyticus*, as well as *V. parahaemolyticus*, *V. harveyi*, *V. vulnificus* and *V. damsel*, are commonly present in aquaculture production systems [28,29]. While the levels of SD_Shr-00004 were far lower in indoor-raised shrimp compared to pond-farmed shrimp, its detectable presence in animals raised in a controlled environment suggests it may be a normal resident in the gut of shrimp. Perhaps much higher abundance, as observed in pond shrimp, could be indicative of a state of dysbiosis. However, since the captured shrimp with high levels of SD_Shr-00004 in this study did not show signs or obvious symptoms of disease, perhaps much higher proliferation of SD_Shr-00004 would be necessary to reach a diseased state or the OTU identified in this study is not a strain of *V. alginolyticus*. Interestingly, Actinobacteria which have recently been reported as a key bacterial phylum in the gut of whiteleg shrimp that allows growth under low salinity conditions and prevents uncontrolled growth of pathogens [12] was on average 50X more abundant in indoor farmed shrimp compared to pond-raised shrimp in this current study.

Together, the results presented in this report indicate that aquaculture practices greatly influence the intestinal bacterial profile of whiteleg shrimp, and further suggest the bacterial communities of this economically important crustacean could in the future be effectively manipulated using diet composition or environmental factors such as water conditions. While future research is necessary to elucidate the mechanisms involved, it also opens the possibility that the same potential for microbiome modulation could exist in other economically important shrimp species, such as the Giant tiger prawn /Asian tiger shrimp (*Penaeus monodon*), Chinese white shrimp /Oriental shrimp /Fleshy prawn (*Fenneropenaeus chinensis*), Brown tiger prawn (*Penaeus penicillatus*), and Banana prawn (*Penaeus merguiensis*).

## Figures and Tables

**Table 1:** Culture tank assignment, diet, time points, average length, and weight of shrimp sampled from the indoor production facility.

Tank	Diet	Time Point (day)	Sample	Average Weight (g)	Average Length (cm)
ST1	Rangen	34	I-R-34 ^a^	1.304	5.2
ST2	Zeigler	34	I-Z-34 ^a^	1.453	5.3
ST3	tSC	34	I-t-34 ^a^	1.248	5.0
STA	Rangen	34	I-R-34 ^b^	1.872	5.8
STB	Zeigler	34	I-Z-34 ^b^	1.278	5.1
STC	tSC	34	I-t-34 ^b^	1.258	5.1
ST1	Rangen	44	I-R-44 ^a^	2.357	6.3
ST2	Zeigler	44	I-Z-44 ^a^	2.381	6.3
ST3	tSC	44	I-t-44 ^a^	2.693	6.6
STA	Rangen	44	I-R-44 ^b^	2.387	6.3
STB	Zeigler	44	I-Z-44 ^b^	2.428	6.4
STC	tSC	44	I-t-44 ^b^	2.039	6.0
ST3	tSC	70	I-t-70 ^a^	7.256	8.8
STC	tSC	70	I-t-70 ^b^	8.868	9.2

The ^a^ or ^b^ are indicative of replicates of the same treatment.

**Table 2:** Partial nutrient content of aquaculture diets ^a^. Values are expressed as percentage (%).

Diet Name	Protein ^b^	Fat	Fiber	Ash	Manufacturer
**Hyper-Intensive 35**	35 (M,P)	7	2	13	Zeigler
**Production 35%**	35 (M,P)	8	3	15	Rangen
**tSC 35**	35 (P)	9	2	12	tSC
**Bumper Crop**	35 (M)	8	3	12	Vimifos

^a^ Formulation of all diets presented in this table are proprietary. ^b^ Primary source of protein ingredients in the diet: marine animal (M), plant (P).

**Table 3:** Relative abundance (%) of main bacterial taxonomic groups in the intestinal tract of whiteleg shrimp raised under two different production systems and from a wild population.

Taxonomic Affiliation	Indoor ^a^	Ponds ^a^	Wild
**Proteobacteria ^#^**	88.6 ± 3.8	51.8 ± 5.4	60.0
Rhodobacteraceae ^#^	84.4 ± 3.8	5.1 ± 5.1	2.7
Vibrionaceae ^#^	0.03 ± 0.01	44.8 ± 5.9	53.5
Other Proteobacteria ^#^	4.2 ± 0.9	1.8 ± 0.7	3.8
**Firmicutes ^#^**	0.7 ± 0.1	36.0 ± 5.9	18.7
**Fusobacteria ^#^**	0.0 ± 0.0	7.9 ± 2.4	3.2
**Bacteroidetes**	2.2 ± 2.0	1.6 ± 0.5	2.2
**Candidatus Saccharibacteria**	2.7 ± 2.2	0.5 ± 0.05	0.0
**Cyanobacteria ^#^**	0.001 ± 0.001	1.6 ± 0.8	7.6
**Actinobacteria ^#^**	3.0 ± 1.1	0.06 ± 0.05	0.2
**Other phyla**	1.6 ± 1.2	0.3 ± 0.2	6.0
**Unclassified bacteria**	1.2 ± 0.3	0.8 ± 0.2	2.3

^a^ Values shown represent mean and standard error of the mean, respectively. ^#^ Means of indoor and ponds samples were statistically different (*P* < 0.05).

**Table 4:** Alpha diversity indices and coverage from gut bacterial communities of whiteleg shrimp raised under two different production systems.

Index	Indoor	Ponds	*P* Value	Wild
Observed OTUs	252 ± 27	177 ± 30	0.0746	422
Ace	1488 ± 327	788 ± 258	0.1082	660
Chao1	724 ± 119	462 ± 129	0.1441	609
Shannon	2.21 ± 0.12	2.13 ± 0.13	0.6403	3.00
Simpson	0.28 ± 0.02	0.28 ± 0.03	0.9682	0.23
Coverage (%)	96.7 ± 0.4	97.9 ± 0.5	0.0685	96.1

**Table 5:** Relative abundance (%) of main Operational Taxonomic Units (OTUs) in the intestinal tract of whiteleg shrimp raised under two different production systems and from a wild population.

OTUs	Indoor ^a^	Ponds ^a^	Wild	Closest Valid Taxon (id%)
**Proteobacteria**				
SD_Shr-00001 ^#^	37.9 ± 4.9	1.5 ± 1.5	0.07	*Phaeobacter piscinae* (98.5%)
SD_Shr-00002 ^#^	23.8 ± 3.1	2.3 ± 2.2	0.2	*Thalassobacter stenotrophicus* (98.5%)
SD_Shr-00004 ^#^	0.02 ± 0.007	26.8 ± 4.6	1.8	*Vibrio alginolyticus* (99.1%)
SD_Shr-00005 ^#^	0.0 ± 0.0	6.4 ± 2.3	46.4	*Photobacterium damselae* (99.1%)
SD_Shr-00006 ^#^	7.8 ± 2.1	0.2 ± 0.1	0.4	*Ruegeria profundi* (99.4%)
SD_Shr-00009 ^#^	2.7 ± 0.8	0.1 ± 0.1	0.09	*Roseovarius pacificus* (99.6%)
**Firmicutes**				
SD_Shr-00003 ^#^	0.0 ± 0.0	28.2 ± 6.2	0.5	*Oceanobacillus iheyensis* (80.9%)
SD_Shr-00008 ^#^	0.0 ± 0.0	3.5 ± 1.1	0.09	*Oceanobacillus iheyensis* (80.6%)
SD_Shr-00046 ^#^	0.003 ± 0.001	0.02 ± 0.004	12.2	*Romboutsia lituseburensis* (98.2%)
**Fusobacteria**				
SD_Shr-00007 ^#^	0.0 ± 0.0	4.4 ± 1.7	0.1	*Propionigenium maris* (96.4%)
SD_Shr-00015 ^#^	0.0 ± 0.0	1.7 ± 0.6	3.0	*Propionigenium modestum* (91.3%)
**Cyanobacteria**				
SD_Shr-00021	0.0 ± 0.0	0.8 ± 0.5	2.5	*Gloeobacter kilaueensis* (86.2%)

^a^ Values shown represent mean and standard error of the mean, respectively. ^#^ Means of indoor and ponds samples were statistically different (*P* < 0.05).

## Supplementary Materials

The following are available online at https://www.mdpi.com/2076-2607/7/4/93/s1.
