# Supplementary material for: Impact of Aquaculture Practices on Intestinal Bacterial Profiles of Pacific Whiteleg Shrimp Litopenaeus vannamei
Source: Microorganisms. 2019 Mar 30;7(4):93. doi: 10.3390/microorganisms7040093 (PMC6518016; doi:10.3390/microorganisms7040093)
Supplement: Supplementary file 1 [file microorganisms-07-00093-s001.zip › microorganisms-458646-s/Landsman et al Supplementary Materials and Methods.docx]

Supplementary Materials and Methods:

It has been well established that variation in nucleotide sequence is not uniform throughout the 16S rRNA molecule, and that certain hypervariable regions are more variable than others. Typically, for instance, V4 and V5 tend to be the least variable, while V1 is amongst the most variable.

The commonly used cutoff of 3% was originally recommended for full length 16S rRNA sequences. While we have no objection to this convention for the analysis of full-length sequences, we disagree that 97% should be used indiscriminately as a cutoff for subregions of the 16S rRNA.

In addition to these considerations, we have also performed in silico experiments using 16S rRNA sequences from validly describing species of well populated genera, such as *Bacillus* and *Prevotella*. We evaluated the percentage dissimilarity amongst species of the same genus for the V1-V3 region (i.e. that datasets consisting of only the V1-V3 region were generated from the full-length sequences, the resulting V1-V3 sequences were aligned, then clustered). The aim was to determine whether species of the same genus had a consistent percentage dissimilarity to each other, which would represent their genus-level cutoff for 16S. For 47 species of *Prevotella* and 115 species of *Bacillus*, both the respective means and medians were approximately 0.13, which is much higher than the value of 0.05 that is commonly used as a genus-level cutoff.

From these considerations, we have made the following determinations:

- If 3% is commonly used as a cutoff for clustering of V4 or V4-V5 regions, which are less variable than V1-V3, then a higher cutoff should be used for V1-V3.
- Since the percentage dissimilarity between species of the same genus for the V1-V3 region is 0.13, then the typical 3% cutoff can justifiably be relaxed.
