# Supplementary material for: Impact of Aquaculture Practices on Intestinal Bacterial Profiles of Pacific Whiteleg Shrimp Litopenaeus vannamei
Source: Microorganisms. 2019 Mar 30;7(4):93. doi: 10.3390/microorganisms7040093 (PMC6518016; doi:10.3390/microorganisms7040093)
Supplement: Supplementary file 1 [file microorganisms-07-00093-s001.zip › microorganisms-458646-s/Supplementary Figure 1-rarefaction.pdf]

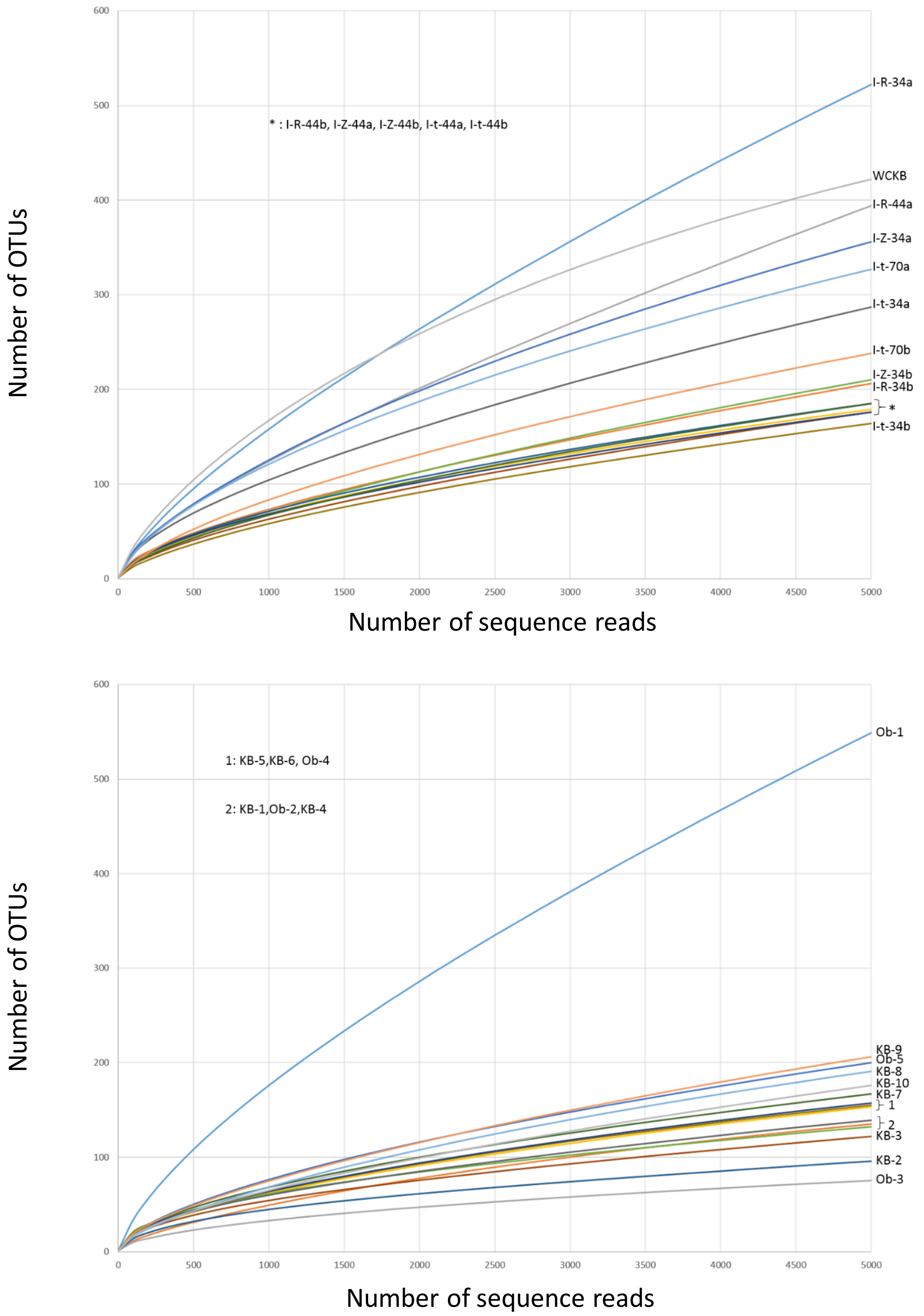

Supplementary Figure 1. Rarefaction curves for sequencing coverage of bacterial 16S rRNA sequences from shrimp intestinal samples.
